# Colorimetric gas detection by the varying thickness of a thin film of ultrasmall PTSA-coated TiO_2_ nanoparticles on a Si substrate

**DOI:** 10.3762/bjnano.8.25

**Published:** 2017-01-24

**Authors:** Urmas Joost, Andris Šutka, Meeri Visnapuu, Aile Tamm, Meeri Lembinen, Mikk Antsov, Kathriin Utt, Krisjanis Smits, Ergo Nõmmiste, Vambola Kisand

**Affiliations:** 1 Institute of Physics, University of Tartu, W. Ostwaldi 1, 50411 Tartu, Estonia; 2 Laboratory of Functional Materials Technologies, Riga Technical University, Paula Valdena 3/7, 1048 Riga, Latvia; 3 Institute of Solid State Physics, University of Latvia, Kengaraga 8, Riga LV-1063, Latvia

**Keywords:** colorimetric gas sensing, *p*-toluenesulfonic acid (PTSA), TiO_2_ nanoparticles

## Abstract

Colorimetric gas sensing is demonstrated by thin films based on ultrasmall TiO_2_ nanoparticles (NPs) on Si substrates. The NPs are bound into the film by *p*-toluenesulfonic acid (PTSA) and the film is made to absorb volatile organic compounds (VOCs). Since the color of the sensing element depends on the interference of reflected light from the surface of the film and from the film/silicon substrate interface, colorimetric detection is possible by the varying thickness of the NP-based film. Indeed, VOC absorption causes significant swelling of the film. Thus, the optical path length is increased, interference wavelengths are shifted and the refractive index of the film is decreased. This causes a change of color of the sensor element visible by the naked eye. The color response is rapid and changes reversibly within seconds of exposure. The sensing element is extremely simple and cheap, and can be fabricated by common coating processes.

## Introduction

The apparent color change in materials induced by structural changes has the potential for applications in sensors with power-free detection and naked-eye readout [1]. Most commonly, the visually perceptible color change of the material is observed in well-ordered structures consisting of building blocks or cavities having uniform size and spacing. It is possible to change or modulate this structural color by changing the interparticle distance by means of a physical or chemical external stimulus. Well-known examples of these materials are opals and inverse opals: three-dimensional photonic crystals where the colors are caused by the periodic variation of the refractive index [2]. Tuneable optical properties in opals are observed by the shift of the Bragg reflection peak of visible light, or by varying the refractive index contrast by liquid or gas infiltration in inverse opals [3–5]. The latter demonstrate an excellent color response with great potential for optical gas detection.

Although a large variety of sensing arrays of periodic well-ordered inverse opal structures has been fabricated, it is still a challenge to fabricate inverse opal structures by straightforward and cost effective large-scale processes. Because of this, it is necessary to improve the fabrication processes of photonic crystals further. Another possibility is the development of completely different color-responsive materials utilising simpler structures or detection principles. Here, we demonstrate an alternative spin-coated thin film of ultrasmall TiO_2_ nanoparticles (NPs) for a colorimetric gas sensor. The functional thin film is extremely simple, consisting of TiO_2_ NPs and the elastic binding agent *p*-toluenesulfonic acid (PTSA) on a Si substrate. It is not necessary to use particles with narrow size distributions of diameters in well-ordered structures, which is a main requirement for other materials providing naked-eye optical gas detection (e.g., inverse opals). The sensing range of the current system is comparable to inverse opal systems. Zhang et al. demonstrated that a silole-infiltrated SiO_2_ inverse opal photonic crystal exhibit a colorimetric response to diethyl ether from 600–1300 ppm, and to petrol ether from 600–1000 ppm (1.95–4.24 mg/L and 2.17–3.62 mg/L, respectively) in air [6]. Lu et al. demonstrated a peak shift in the extinction spectrum of approximately 15 nm for ethanol vapor concentrations from 0–10000 ppm in metal-organic framework containing colloidal silica crystals [7]. The current sensor system, which is simpler and also cheaper to fabricate, gives a peak shift of approximately 4 nm in this concentration range.

The color of TiO_2_ NPs thin films changes here after the absorption of volatile organic compounds (VOCs) into the PTSA binding agent between the NPs and the subsequent swelling of the film. Thus, the film thickness and the optical path length of the light in the film are changed. This varies the interference wavelengths of light reflected by the substrate and the thin film. The change of thickness and the apparent color of the functional TiO_2_ NP thin films is rapid, and also changes reversibly within seconds of exposure. The sensor exhibits a gradual color change from yellow to green/blue upon exposure, and also a selectivity to different VOCs with the highest response (i.e., the largest shift of interference maxima and minima) to isopropanol. In the present work results obtained using one typical thin film are presented. However, experiments were repeated with several thin films all showing similar behavior.

## Experimental

Titania NPs were synthesised using a method described by Scolan and Sanchez [8] with slightly modified parameters [9–10]. Commercially available titanium(IV) butoxide (Sigma-Aldrich, reagent grade), *p*-toluenesulfonic acid (PTSA) (Sigma-Aldrich, reagent plus), acetylacetone (acac) (Sigma-Aldrich, reagent plus), butanol (Sigma-Aldrich) and deionised water were used as precursors. The solvent (butanol) was dried using CaH_2_ and distilled before use. The molar ratio between PTSA and titanium(IV) butoxide was set to 0.2, that between acac and titanium(IV) butoxide was set to 3, and that between water and titanium(IV) butoxide was set to 10. In a typical synthesis 9.0 g of titanium(IV) butoxide was dissolved in 30.0 g of butanol, 7.953 g of acac was added. A solution of PTSA was prepared by dissolving 1.2072 g of PTSA in 5.6087 g of DI water. An amount of 5.6769 g of the solution was added dropwise to the reaction mixture. The reaction was carried out overnight under reflux conditions. The nanoparticles were washed twice with methanol using centrifugation at 12000*g* for 1 h. The synthesis was optimized to obtain ultrasmall nanoparticles (roughly 3 nm in diameter) to attain a high sample surface area. Our modified synthesis protocol had a NP yield more than 50% after washing.

Thin films based on TiO_2_ nanoparticles were prepared from the NP colloidal solution (5.9% by mass in ethanol) by spin coating on Si(100) substrates in ambient atmosphere. The substrates were cleaned prior to coating with ethanol to remove small dust particles. The rotation frequency during spin coating was 3000 rpm and coating time was 0.5 min. The obtained NP-based precursor films were aged at room temperature under ambient conditions for four days. The purpose of this ageing was to allow the remaining solvent to evaporate slowly, in order to prevent the cracking of the films. After ageing, the films consisted of PTSA-covered TiO_2_ NPs. PTSA could have been removed by annealing, but the aged films were not heated up, to maintain the ability of the films to swell.

A Sopra GES-5E variable angle spectroscopic ellipsometer was used to determine the thickness (*d*) and optical properties (refractive index *n*, absorption coefficient *k*) of the films using the “Winelli II” software. Film thickness and optical constants were determined from the ellipsometric parameters tan ψ and cos Δ [11]. All the main parameters (*d*, *n*, and *k*) were obtained using a Levenberg–Marquardt non-linear regression algorithm. Ellipsometric measurements were generally performed at incidence and reflectance angles of 75°. TiO_2_ films made from NPs were modelled as homogeneous mixtures of supposedly dense materials, with the addition of voids for the adjustment of *n* and *k*. The optical properties of the thin films were examined in air under ambient conditions. The optical constants given here are those measured at 633 nm wavelength.

Using ellipsometry, the refractive index of the film was modelled between 1.2–4.5 eV at a wavelength of 633 nm. The three-layer stack (silicon substrate/silicon oxide/TiO_2_ film/mixture of TiO_2_ and voids) was used for modelling and fitting both thickness and refractive index using the standard three-angle data sets.

X-ray photoelectron spectroscopy (XPS) was used for investigating the chemical state and elemental composition of the NP-based films after different treatments. XPS measurements were conducted using a surface station equipped with an electron energy analyzer (SCIENTA SES 100) and a non-monochromatic twin anode X-ray tube (Thermo XR3E2), with characteristic energies of 1253.6 eV (Mg Kα_1,2_ FWHM 0.68 eV) and 1486.6 eV (Al Kα_1,2_ FWHM 0.83 eV). All XPS measurements were conducted under ultra-high vacuum (UHV) conditions. The binding energy scales for the XPS experiments were referenced to the binding energy of Ti^4+^ 2p_3/2_ (458.6 eV). To estimate the overall atomic concentrations of different compounds and elements, the average matrix relative sensitivity factors (AMRSF) procedure [12] and the transmission function of our instrument were used. The raw data were processed using the Casa XPS software [13]. Data processing involved removal of Kα and Kβ satellites, removal of the background and fitting of the components. Background removal was carried out using Tougaard background; for fitting, the Gauss–Lorentz hybrid function was used (GL 70, Gauss 30%, Lorentz 70%). However, the absolute amounts of different compounds and elements have to be considered cautiously, and are given to outline trends and estimates only. Due to the possible deviation of the surface region from chemical homogeneity in the working range of photoelectron spectroscopy (surface region with a thickness of up to three electron mean free paths), some signals might be amplified or suppressed.

For calculation of XPS spectra, the GPAW program [14–15] was used, which is a real-space uniform grid-based all-electron DFT code implemented in the projector augmented-wave (PAW) [16]. The ground-state energy was calculated with the geometry obtained from Gaussian 09 calculations using PBE functionals and a grid spacing of *h* = 0.16 Å. For C, O, S and N atoms core electrons were excited one by one to obtain the excited-state energies using the same calculation parameters as for the ground state, and adding a local combination of atomic orbital modes.

The crystalline phases of the titania NPs were examined by measuring room-temperature Raman spectra of the films prepared on a fused silica substrate using a Renishaw micro-Raman set-up equipped with a 514 nm continuous mode argon ion laser, of approximate spectral resolution 1.5 cm^−1^.

The AFM measurements, with the purpose of investigating the thickness of the films before and after exposure to VOCs, were conducted using a Veeco AFM. Typically, the tapping mode was utilized in order to provide an optimal performance. OTESPA AFM tips (manufactured by Bruker) were used. To measure thickness of films, they were scratched with stainless steel tweezers and the step height of the scratch was measured. To ensure that only the film was scratched away (and not the substrate) scratching was carried out using different forces; the results were similar. The position of the bottom of the scratch was measured from three different places and so was the surface of the film; the measurements were averaged. In case of AFM measurements the films were exposed to ethanol vapor by placing them in a Petri dish and dropping ethanol in the vicinity of the sample.

Optical transmission and reflection measurements were conducted with a Cary 5000 (UV–vis–NIR) spectrometer (Agilent Technologies) during the exposure of the film in a gas flow cell. The spectrometer was equipped with an internal diffuse reflectance accessory (DRA). The DRA was configured so that both diffuse reflectance and specular reflectance were measured. The flow cell was attached to the measurement window of the DRA and was fastened securely.

**Figure 1:**
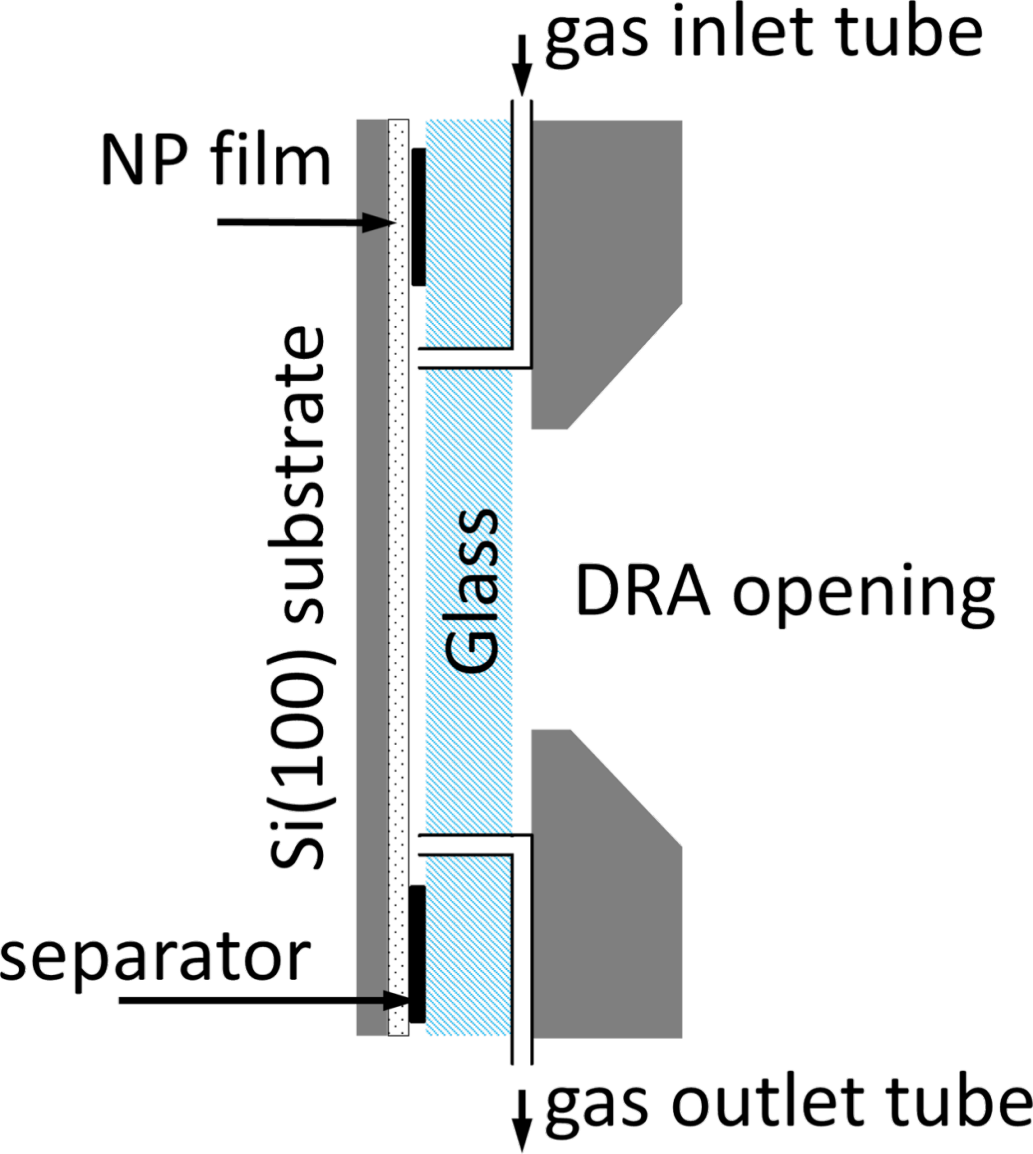
Construction of the flow cell for reflectance measurements.

The flow cell (Figure 1) was constructed using 1 mm thick soda-lime glass, a 150 µm thick separator, and a silicon monocrystal plate covered with the TiO_2_ NPs film. The gas inlet and outlet were introduced through the glass plate. The separator was placed between the TiO_2_ NP-covered silicon monocrystal, and the monocrystal was sealed hermetically to the glass plate using epoxy resin, forming a flow through the cell. VOC/air mixtures were prepared by using an automatic pipette and dropping the required amounts of the specific VOC to a 2.5 L glass jar through a 3 mm opening in the cap, the jar was equipped with a magnetic stirrer to homogenize the gas mixture. Before filling the syringe with 20 mL of the gas mixture the gas was stirred for 3 min. During measurements, 20 mL of air with certain amounts of VOC vapor was injected with a syringe through the cell and the reflectance spectra were measured. After measurements, the cell was purged with 200 mL clean air and the measurements with every VOC were repeated twice and then the next VOC/air mixture was measured.

The hydrodynamic diameter and the respective size distribution of the NPs were measured in ethanol using dynamic light scattering (DLS, Zetasizer Nano ZSP, Malvern Instruments). The microstructural features of the nanoparticles were studied by a transmission electron microscope (TEM, Tecnai G20, FEI) operated at 200 kV.

## Results and Discussion

**Figure 2:**
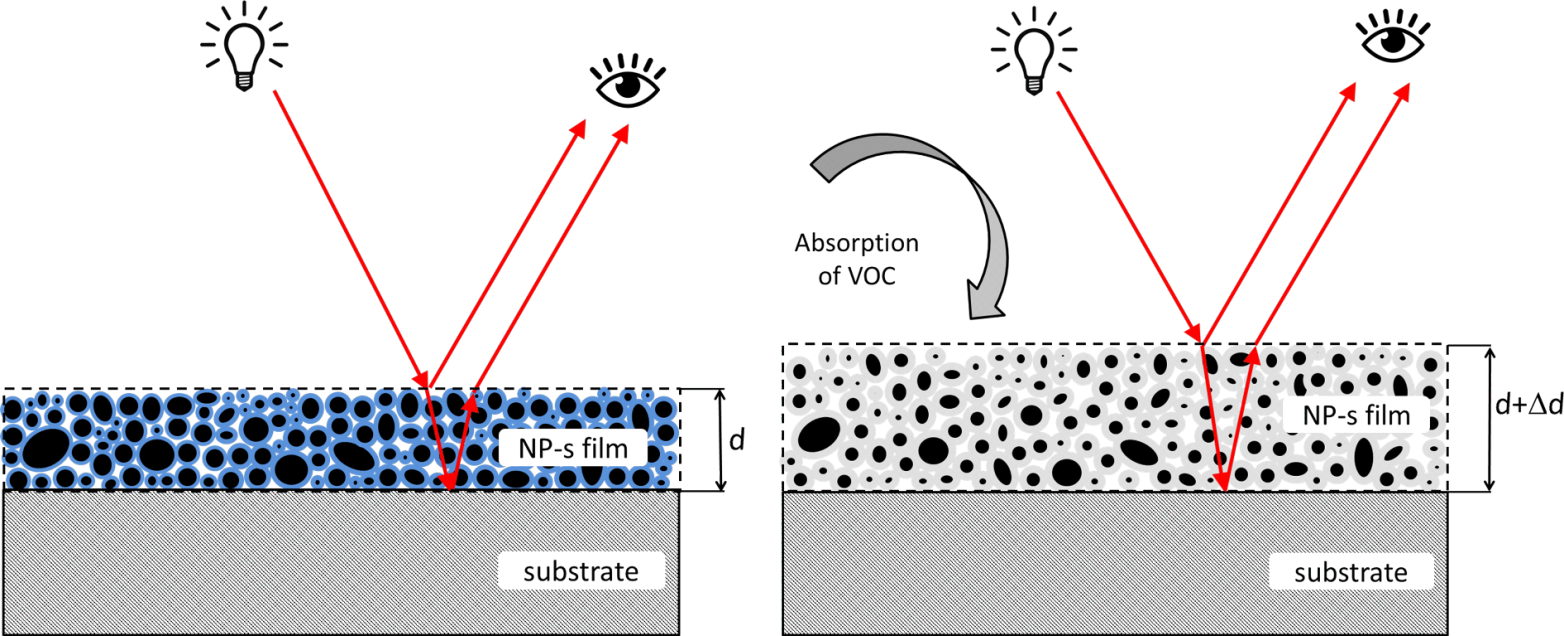
Working principle of the sensor element: the light reflected from the surface of the film and from the film/substrate interface will interfere. Left: situation before absorption of VOCs; right: situation after absorption of VOCs. The optical path length is changed by the absorbed VOC molecules due to the twofold mechanism: (i) physical swelling of the film (*d*→*d* + Δ*d*) and (ii) decrease of the refractive index of the film (*n*→*n* − Δ*n*). Black circles mark NPs; blue area on the left panel represents PTSA; and grey area on the right panel represents a mixture of PTSA and VOC.

**Figure 3:**
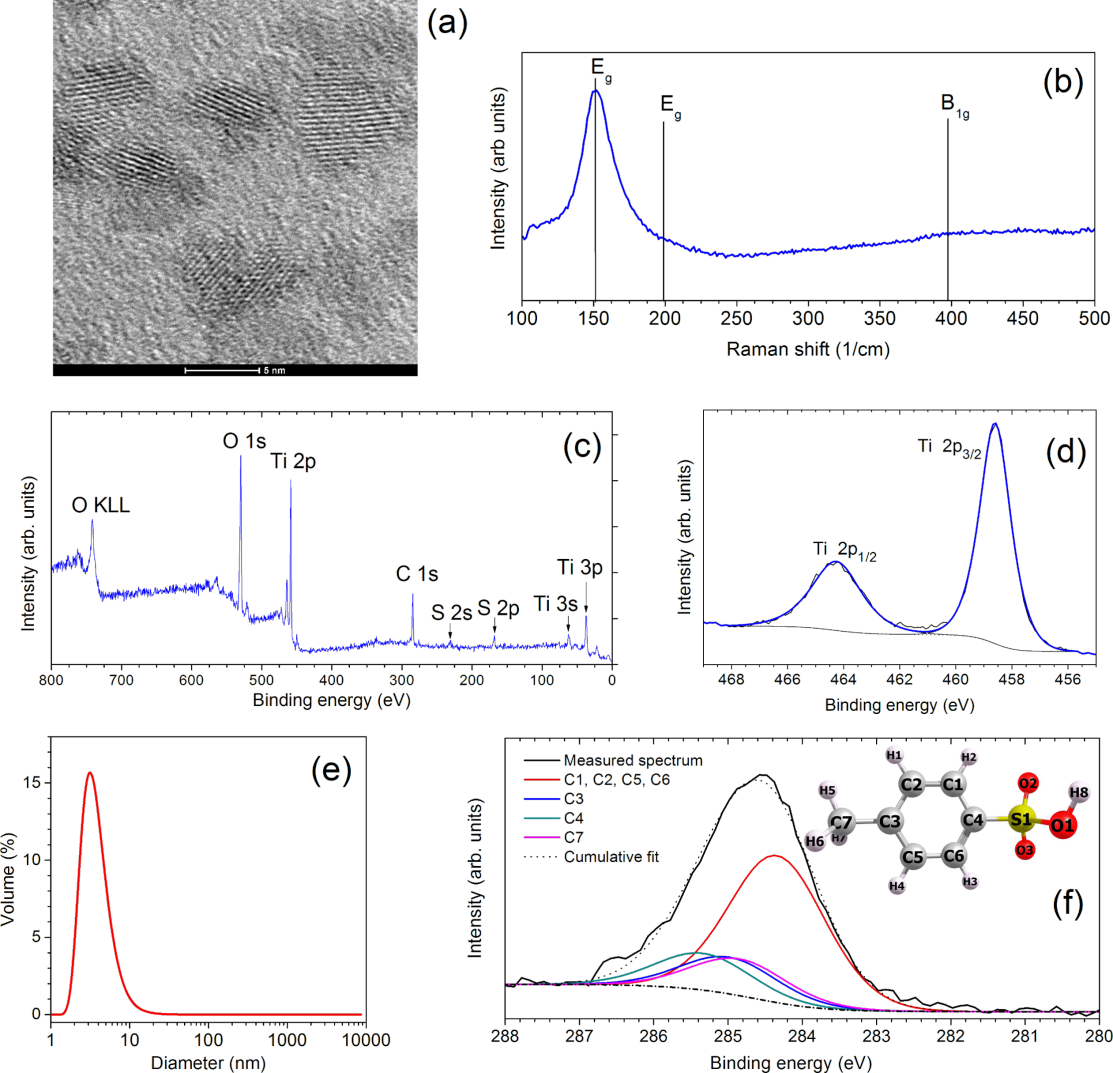
Characterization of the synthesized NPs and their thin films bound with PTSA. (a) TEM images of TiO_2_ nanoparticles; (b) Raman spectrum of the NP-based thin film showing the existence of the anatase crystal phase (E_g_ band at 151 cm^−1^); (c) XPS overview spectrum demonstrating the presence of titanium, oxygen, carbon and sulfur in the NP-based thin film; (d) Ti 2p photoemission lines typical for Ti^4+^ do not show the presence of reduced titanium ions (Ti^3+^) in TiO_2_; (e) hydrodynamic size distribution of the NPs (measured by DLS in colloidal dispersion); (f) measured XPS spectrum of the C 1s region and calculated XPS spectra for PTSA including sub-bands. Locations of the respective atoms in the PTSA molecule are also shown (inset).

Optical gas sensors based on porous Bragg stacks utilize the phenomenon of analyte vapor being absorbed in the pores of the stack, which changes the effective refractive index of the individual layers [1,17–18]. Our objective was to demonstrate a simpler and more cost-effective system utilizing the gas absorption in films and the subsequent swelling of the films, which changes the interference maxima and minima of light reflected from the surface of the film and from the film/substrate interface, as schematically demonstrated in Figure 2. The functional thin film is extremely simple, consisting of TiO_2_ NPs (the mean size of 3 nm was measured by TEM and DLS, Figure 3a and Figure 3e) and the binding agent PTSA on a Si substrate. It is a colorimetric gas sensor based on a single-layer NP film (Figure 2), where the NPs in the film are distributed rather randomly. PTSA absorbs VOCs and the thickness of the film increases (also the refractive index decreases) changing the interference color. Neither VOCs nor PTSA react with the TiO_2_ NPs.

Raman measurements demonstrated that the NPs bound into the film were in the anatase crystal phase (see Figure 3b) and had good crystallinity. The main anatase Raman band (E_g_) had slightly shifted from 144 cm^−1^ (the typical value for anatase TiO_2_ powders) to 151 cm^−1^. Such a shift has been explained by a small diameter (some nanometers) or the nonstoichiometry of the nanoparticles [19]. As will be demonstrated, the measured XPS spectra show a stoichiometric composition of the NP material. Therefore in our case, the shift of the Raman peak to 151 cm^−1^ was caused by the small mean diameter of the nanoparticles (ca. 3 nm).

XPS was used to characterize the surface region of the NP-based film. From the overview spectrum (Figure 3c) the presence of oxygen, titanium, carbon and sulfur can be seen; no other elements were detected on the film surface. The Ti 2p spectrum corresponds well to literature data [20–21] of TiO_2_, and fits well with two splines (2p_3/2_ and 2p_1/2_) demonstrating that only Ti^4+^ (and no Ti^3+^) is present on the surface (Figure 3d). The hydrodynamic size of the particles was measured in colloidal dispersion with DLS. The mean size of the particles was ca. 3 nm, as can be seen from Figure 3e, and the size distribution was rather narrow (standard deviation 2.1 nm). The particle suspension in ethanol was stable over time, and the DLS spectra did not show any sign of aggregates and agglomerates after one year. Because of the very small mean size of the nanoparticles sedimentation was also excluded.

The experimental C 1s XPS spectrum (Figure 3f) demonstrates a strong C 1s peak. To confirm the hypothesis that the carbon in the films is connected to PTSA (*p*-toluenesulfonic acid), the experimental C 1s spectrum was compared to the calculated C 1s spectrum of PTSA. Therefore, the geometry *p*-toluenesulfonic acid in the gas phase was first optimized with Gaussian 09 [22] using the density functional theory (DFT)-based exchange and correlation functional Perdew–Burke–Ernzerhof (PBE) [23–24], and cc-pvtz basis sets [25–28]. The optimized geometry was then used to calculate the XPS spectra. These were calculated in GPAW using the full core–hole transition potential approach [29], as described in the Experimental section. The good agreement of the measured C 1s XPS spectrum with the calculated XPS C 1s spectrum of PTSA demonstrates that carbon in the films originates from PTSA. Also, the position of the S 2p photoemission line in the overview spectrum (168.2 eV, Figure 3c) is consistent with the sulfonic acid group [30]. This demonstrates that the nanoparticles are covered with PTSA molecules, yielding specific surface properties such as polarity and affinity towards the specific compounds.

**Figure 4:**
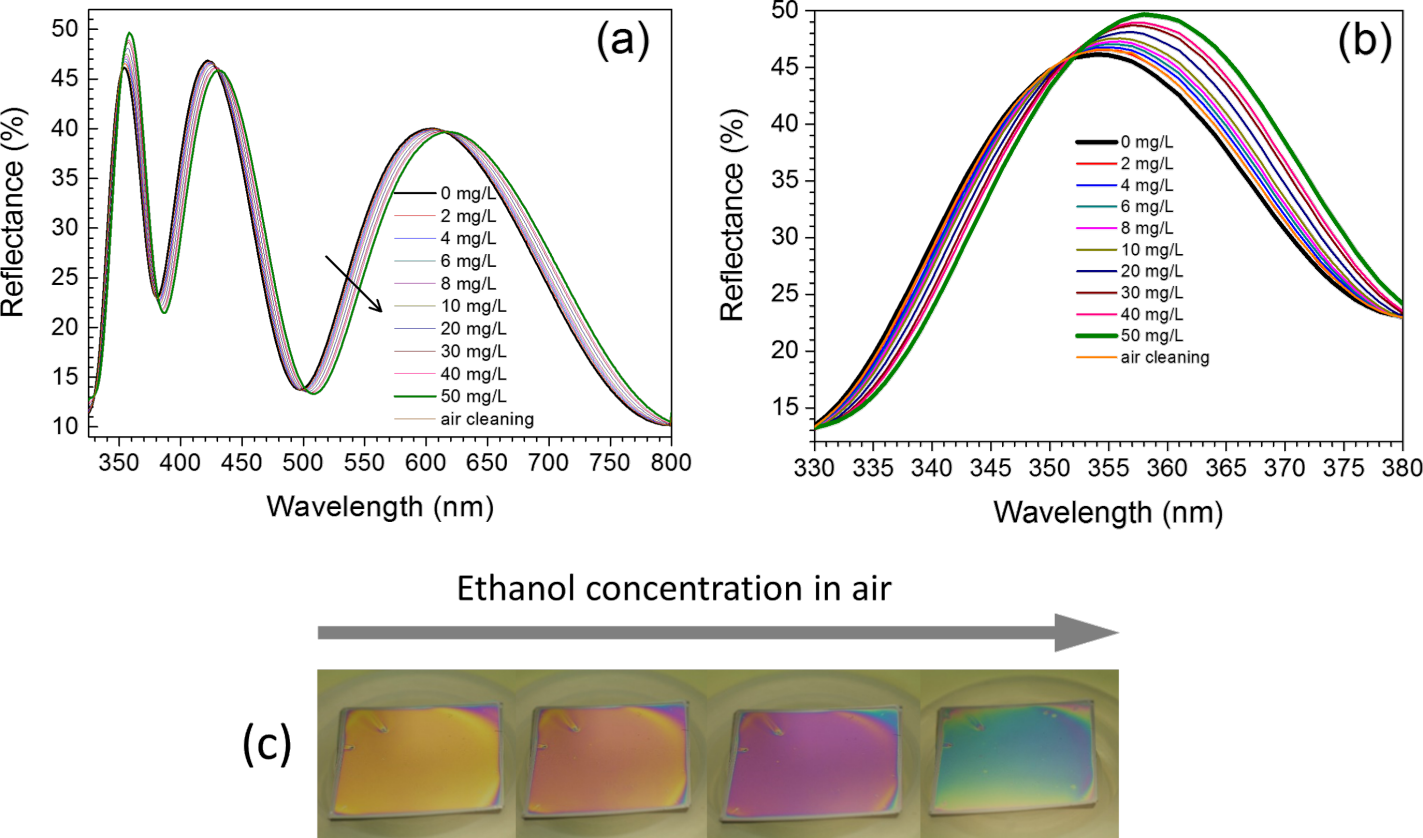
(a) UV–vis reflectance spectra of the film exposed to different ethanol concentrations and cleaned sample. (b) Short-wavelength region of the previous spectrum at larger scale; (c) Change of the color of the film with increase of the ethanol concentration (no ethanol, ethanol/water 10:90, ethanol/water 50:50, ethanol/water 90:10).

The vapor-sensing ability of the prepared TiO_2_ NP-based film was investigated by measuring UV–vis reflectance spectra of the film exposed to different ethanol concentrations (Figure 4a and Figure 4b). A clear and systematic wavelength shift as a result of an increase of the ethanol concentration can be observed in the interference maxima and minima. After each measurement the sample was purified with air and a control measurement was performed. All control spectra were identical with the initial spectrum measured before the start of the experiments. This demonstrates that ethanol was completely removed after cleaning with air, and the sample properties (including initial thickness) were recovered. The shift of the interference maxima and minima is sufficiently large to give optical responses visible by the naked eye (Figure 4c). When the ethanol concentration is increased, the color of the films changes from yellow to green/blue. The color change was extremely rapid, occurring within seconds of exposure, which could be explained by the presence of open pores in the thin film, thus enhancing the gas diffusion rate. To monitor the color change, the film was exposed to ethanol vapors by placing different concentrations of ethanol/water mixtures into the Petri dish located near the sample.

In the TiO_2_ NP-based thin films, the incident light is, in part, reflected back from the air/film interface and, in part, from the substrate surface and the light waves interfere. The position of the maxima and minima of interference depends on the optical path length difference of the light reflected from the air/film and film/substrate interfaces. This difference is determined by film thickness and refractive index [11]. When VOC molecules are absorbed in NP-based film, swelling occurs, and the optical path length of the light increases. As a result, the positions of the interference maxima and minima shift in the reflection spectrum, and also the apparent color of the film changes. Swelling of the film was demonstrated by AFM thickness measurements, where the thickness of the film, i.e., the height between the substrate and the film surface was measured before and after VOC exposure. Additionally, changes in the surface structure of the films under VOC exposure are possible. However, reflectance modifications in Figure 4 are typical for films with increasing thickness and therefore we can neglect significant changes in the surface structure under VOC exposure.

**Figure 5:**
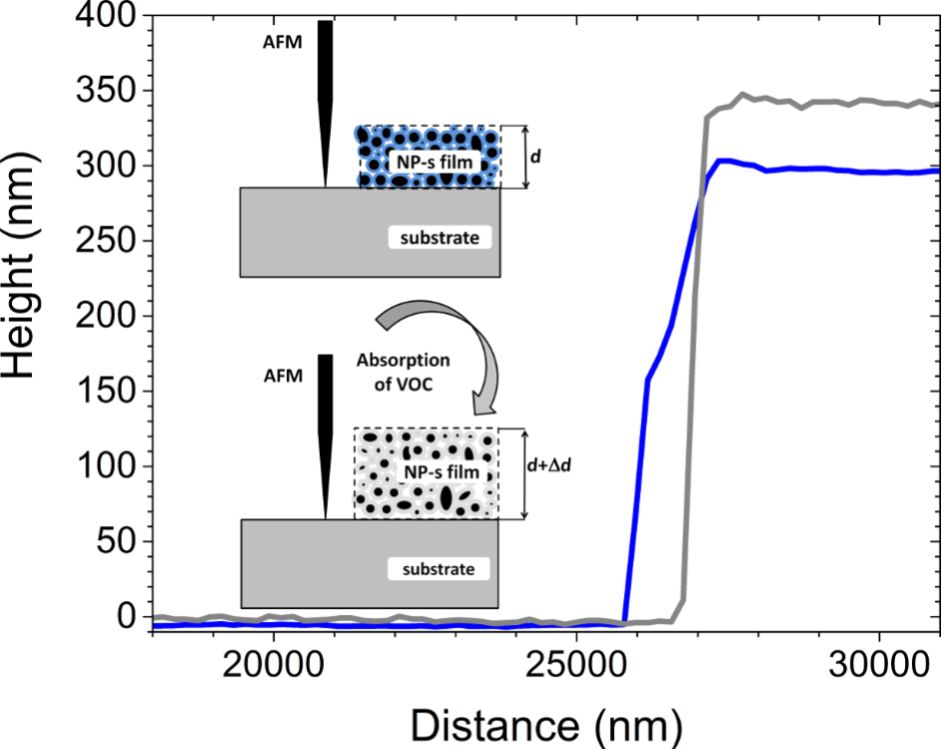
Left: schematic diagram of height profile measurements before and after absorption of ethanol. Right: AFM height profile of the NP-based thin film before (blue) and during exposure (grey) to ethanol vapors.

Figure 5 shows the swelling of the film during exposure to the analyte gas (ethanol). The film thickness was evaluated to be ca. 305 nm before exposure to ethanol vapors and ca. 345 nm during exposure, i.e., the measured swelling is about 10%. This swelling is related to the absorption of VOCs (not adsorption). Since AFM is used in tapping mode, it would not be able to show an adsorption layer of gas on the top of sample. However, beside absorption also adsorption of VOCs can take place and the coexistence of adsorption and absorption in our NP-based film deserves further research.

The key point of our approach is that the optical path length is changed by absorbed gas molecules due to a double mechanism: (i) physical swelling of the film (*d*→*d* + Δ*d*) and (ii) decrease of the refractive index of the film (*n*→*n* − Δ*n*). Corroborating the AFM studies, ellipsometry measurements showed a clear change of thickness and also a change of the refractive index during exposure to ethanol. The ellipsometry results showed a swelling of the film of roughly 6% and, at the same time, a decrease of the refractive index from 2.06 to 1.99, i.e., 3% measured at 365 nm, and from 1.87 to 1.81, i.e., also 3% measured at 633 nm. A decrease of the refractive index during swelling is understandable, taking into account the refractive index of ethanol (1.36 at 633 nm).

**Figure 6:**
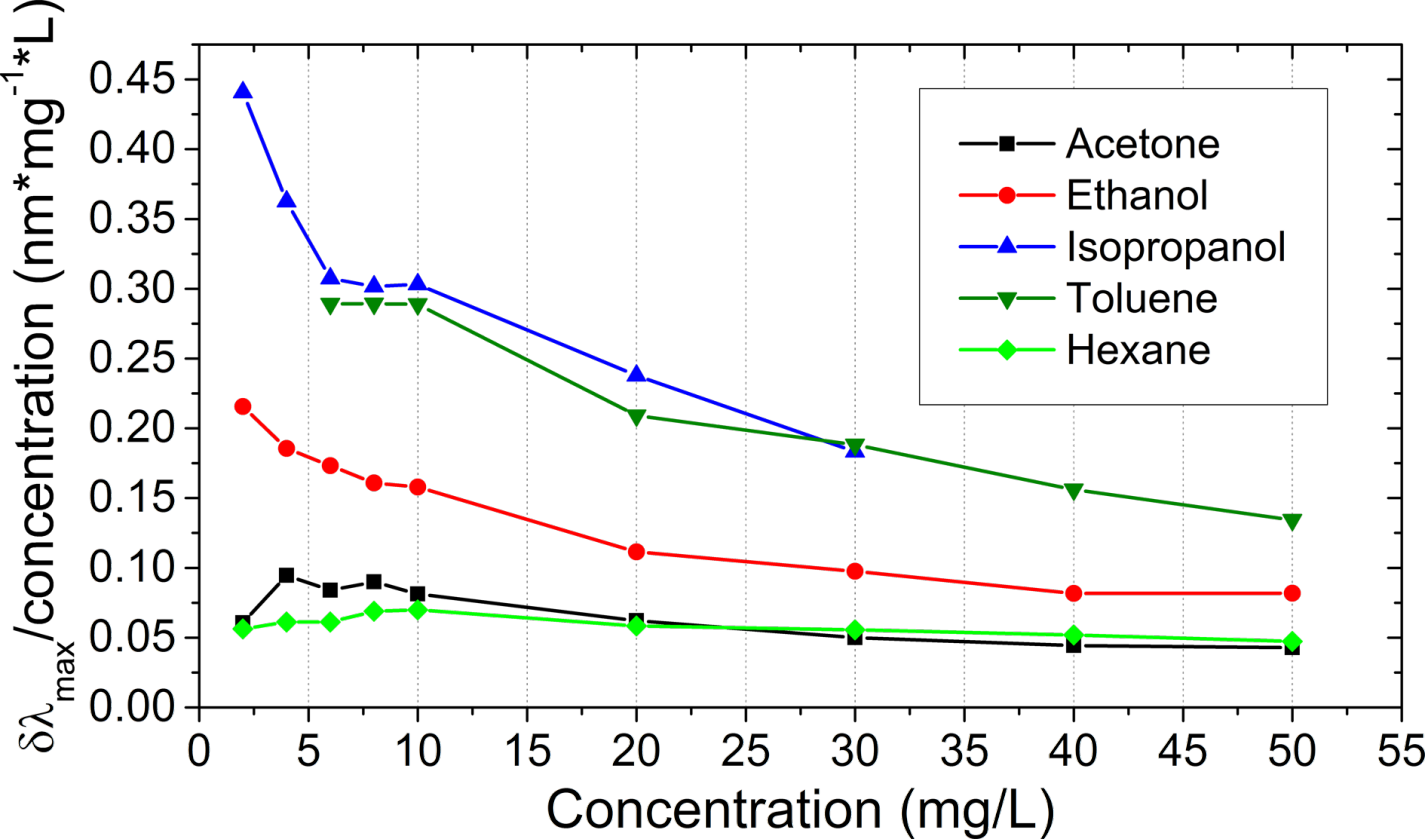
Optical response of the TiO_2_ NP-based thin film towards different VOCs (i.e., dependence of the shift of the reflection maximum on the VOC concentration).

The selectivity to different gases can be achieved by surface functionalization of the nanoparticles, which was previously utilized both in localized surface plasmon resonance (LSPR) devices and porous Bragg stacks [17]. As mentioned before, the NPs in our samples are covered with PTSA. This functional coating ensures selectivity and different responses to different VOCs. Figure 6 shows that the proposed NP film-based sensor element is more responsive to isopropanol and toluene, and the response to hexane is much smaller. Toluene probably interacts strongly with the aromatic ring in PTSA, and isopropanol is probably more compatible with the polar part of the molecule, due to the ability to form hydrogen bonding. Different sensitivities to different VOC vapors can potentially be used to discriminate between various substances using multiple sensor arrays with different functional coatings. The presented sensor element is very cheap to manufacture and sensitive enough to give optical responses visible by the naked eye. The response is fast, and no differences could be noticed in the spectra acquired right after the introduction of the air/VOC mixture and the spectra taken 10 s later. The visible response is instantaneous after the introduction of a VOC vapor.

## Conclusion

Here, we proposed a simple and cost-effective colorimetric gas sensing system utilizing the absorption of the analyte into a PTSA-modified thin film based on TiO_2_ NPs. Volatile organic compounds absorb into the PTSA surrounding the nanoparticles, and subsequently cause a significant swelling of the films. Thus, the optical path length in our NP-based film is changed by the absorbed gas molecules using a double mechanism: (i) physical swelling of the film (*d*→*d* + Δ*d*) and (ii) decrease of the refractive index of the film (*n*→*n* − Δn). Due to this reason, in UV–vis reflectance spectra of the NP-based film exposed to different ethanol concentrations, a clear and systematic wavelength shift of the interference maxima and minima was observed as a result of the increase in ethanol concentration. It was also demonstrated that after cleaning the sample with air, the effect was fully reversible: Ethanol was completely removed, and the optical properties of the sample were recovered. Overall, the proposed colorimetric sensor element is very simple and cheap to manufacture, and is sensitive enough to give optical responses to analyte gases visible by the naked eye.

## References

[1] Burgess I B, Lončar M, Aizenberg J (2013). J Mater Chem C.

[2] Hong W, Li H, Hu X, Zhao B, Zhang F, Zhang D (2012). Chem Commun.

[3] Singleton T A, Burgess I B, Nerger B A, Goulet-Hanssens A, Koay N, Barrett C J, Aizenberg J (2014). Soft Matter.

[4] Hong W, Chen Y, Feng X, Yan Y, Hu X, Zhao B, Zhang F, Zhang D, Xu Z, Lai Y (2013). Chem Commun.

[5] Diao Y Y, Liu X Y, Toh G W, Shi L, Zi J (2013). Adv Funct Mater.

[6] Zhang Y, Qiu J, Gao M, Li P, Gao L, Heng L, Tang B Z, Jiang L (2014). J Mater Chem C.

[7] Lu G, Farha O K, Kreno L E, Schoeneker P M, Walton K S, van Duyne R P, Hupp J T (2011). Adv Mater.

[8] Scolan E, Sanchez C (1998). Chem Mater.

[9] Joost U, Saarva A, Visnapuu M, Nõmmiste E, Utt K, Saar R, Kisand V (2014). Ceram Int.

[10] Joost U, Juganson K, Visnapuu M, Mortimer M, Kahru A, Nõmmiste E, Joost U, Kisand V, Ivask A (2015). J Photochem Photobiol, B: Biol.

[11] Fujiwara H (2007). Spectroscopic Ellipsometry Principles and Applications.

[12] Seah M P, Gilmore I S, Spencer S J (2001). J Electron Spectrosc Relat Phenom.

[13] (2000). CasaXPS.

[14] Enkovaara J, Rostgaard C, Mortensen J J, Chen J, Dułak M, Ferrighi L, Gavnholt J, Glinsvad C, Haikola V, Hansen H A (2010). J Phys: Condens Matter.

[15] Mortensen J J, Hansen L B, Jacobsen K W (2005). Phys Rev B.

[16] Blöchl P E (1994). Phys Rev B.

[17] Bonifacio L D, Puzzo D P, Breslav S, Willey B M, McGeer A, Ozin G A (2010). Adv Mater.

[18] Bonifacio L D, Ozin G A, Arsenault A C (2011). Small.

[19] Li Bassi A, Cattaneo D, Russo V, Bottani C E, Barborini E, Mazza T, Piseri P, Milani P, Ernst F O, Wegner K (2005). J Appl Phys.

[20] Hashimoto S, Tanaka A (2002). Surf Interface Anal.

[21] Dhayal M, Sharma S D, Kant C, Saini K K, Jain S C (2008). Surf Sci.

[22] (2009). Gaussian 09.

[23] Perdew J P, Burke K, Ernzerhof M (1996). Phys Rev Lett.

[24] Ernzerhof M, Perdew J P (1998). J Chem Phys.

[25] McQuarrie D A (2008). Quantum Chemistry.

[26] Scuseria G E (1999). J Phys Chem A.

[27] Fantin P A, Barbieri P L, Neto A C, Jorge F E (2007). J Mol Struct: THEOCHEM.

[28] Weigend F, Ahlrichs R (2005). Phys Chem Chem Phys.

[29] Ljungberg M P, Mortensen J J, Pettersson L G M (2011). J Electron Spectrosc Relat Phenom.

[30] Nasef M M, Saidi H (2006). Appl Surf Sci.

